# Local mortality estimates during the COVID-19 pandemic in Italy

**DOI:** 10.1007/s00148-021-00857-y

**Published:** 2021-06-19

**Authors:** Augusto Cerqua, Roberta Di Stefano, Marco Letta, Sara Miccoli

**Affiliations:** 1 Department of Social Sciences and Economics, Sapienza University of Rome, Rome, Italy; 2 Department of Statistical Sciences, Sapienza University of Rome, Rome, Italy; 3 Department of Methods and Models for Economics, Territory and Finance, Sapienza University of Rome, Rome, Italy

**Keywords:** COVID-19, Coronavirus, Local mortality, Italy, Machine learning, Counterfactual building, C21, C52, I10, J11

## Abstract

Estimates of the real death toll of the COVID-19 pandemic have proven to be problematic in many countries, Italy being no exception. Mortality estimates at the local level are even more uncertain as they require stringent conditions, such as granularity and accuracy of the data at hand, which are rarely met. The “official” approach adopted by public institutions to estimate the “excess mortality” during the pandemic draws on a comparison between observed all-cause mortality data for 2020 and averages of mortality figures in the past years for the same period. In this paper, we apply the recently developed machine learning control method to build a more realistic counterfactual scenario of mortality in the absence of COVID-19. We demonstrate that supervised machine learning techniques outperform the official method by substantially improving the prediction accuracy of the local mortality in “ordinary” years, especially in small- and medium-sized municipalities. We then apply the best-performing algorithms to derive estimates of local excess mortality for the period between February and September 2020. Such estimates allow us to provide insights about the demographic evolution of the first wave of the pandemic throughout the country. To help improve diagnostic and monitoring efforts, our dataset is freely available to the research community.

**Supplementary Information:**

The online version contains supplementary material available at 10.1007/s00148-021-00857-y.

## Introduction

The COVID-19 pandemic is a complex and constantly evolving phenomenon that is affecting the entire world heterogeneously. The disease caused by the spread of this new form of coronavirus has rapidly propagated worldwide, affecting countries with different timing and intensity (Ceylan 2020). Italy was the first country in Europe to be hit by COVID-19, and, to date, it ranks among the countries with the highest fatality toll. Given the diverse geographical spread of the pandemic, local estimates provide an important tool to document the spread of COVID-19. However, the official data on the death toll of COVID-19 are scarce at the local level, and, when available, they are likely to suffer from substantial underreporting (Ghislandi et al. 2020).^1^ Besides, such measurement error is likely to be heterogeneous even within countries (Leon et al. 2020), and this inaccuracy may lead to incorrect beliefs about how COVID-19 spreads across space and over time. A valid alternative for estimating the number of deaths caused by the pandemic (directly or indirectly) consists of considering the number of daily certified all-cause deaths (deaths from any cause, not only those related to coronavirus).^2^ In particular, using historical data on the number of daily certified deaths to estimate the number of daily deaths in the absence of the pandemic could vastly reduce the uncertainty associated with COVID-19 official data, especially at a disaggregated geographical level. This study applies this approach to Italy.

As the pandemic has affected the entire country, it is not feasible to use the most common counterfactual approach that compares treated and non-treated municipalities within the country. In this unusual setting, the benchmark approach to estimate excess mortality — which we will call the “intuitive” approach — adopted by several national and international institutions, and employed in many scientific works (see Section 2), consists of comparing the actual number of cumulative all-cause deaths in 2020 with the numbers observed in the past for the same municipality.^3^ Stated differently, it is a before–after analysis of the changes between post-pandemic mortality and the pre-pandemic average over the past year(s). Yet, being simply an unconditional average, this comparison does not employ any covariates, nor indeed any model, and may be sensitive to outliers and other data issues. Therefore, it may be considered as an excessively coarse approach to generate excess mortality figures, especially when employed at a fine geographical scale.

This paper, instead, makes use of machine learning (ML) and municipality-level panel data from the recent past (from 2015 to 2019) to build the counterfactual scenario. By applying ML algorithms on all-cause deaths’ data, we credibly estimate excess mortality in Italian municipalities (local administrative units, LAU) from February 21, 2020 (the official date of the first coronavirus cluster in Italy), to September 30, 2020.^4^ To this aim, we compare the official number of cumulative all-cause deaths for the period February 21 to September 30, 2020, with an estimate of the number of cumulative deaths over the same period in an “ordinary” situation, i.e., in the counterfactual situation without the pandemic. We then consider the difference between observed mortality and our counterfactual predictions as the cumulative number of excess deaths, which are very likely to be due, either directly or indirectly, to COVID-19. Differently from the intuitive approach, however, our counterfactual scenario is not just a pre-pandemic mortality average but rather an ML-generated prediction for each municipality, generated using only pre-pandemic information, of an alternative, no-pandemic, “business-as-usual” mortality scenario.

It is important to note that we estimate the gross excess mortality due to COVID-19, i.e., the number of deaths due directly to COVID-19 infections as well as deaths due to the collateral effects of the lockdown. Such collateral effects have lowered the likelihood of dying from some causes such as road and workplace accidents, pollution-related diseases, or criminal activities and increased the likelihood of dying owing to the unprecedented stress on the public health system (e.g., severe delays in the hospitalization process).

We report estimates obtained using three estimation approaches: intuitive, ML, and a classical linear method. The intuitive approach sketched above is used exclusively as a benchmark to evaluate the performance of the ML techniques. We then employ three ML algorithms to estimate excess mortality for all municipalities. ML techniques allow predicting mortality trends by “training data,” i.e., by learning from past information, evaluating out-of-sample model performance on unseen “testing data”, and finally comparing predicted and actual values to derive excess mortality figures. Specifically, we employ the least absolute shrinkage and selection operator (LASSO), random forest, and stochastic gradient boosting. Finally, for the sake of comparison, we also test the performance of a straightforward OLS regression.

We show how all these methods outperform, on average, the intuitive approach adopted by Italian institutions with mean squared error (MSE) predictive gains of up to 17.8% and especially sizable improvements in smaller municipalities. Thanks to the higher accuracy of data-driven methods, we improve the estimates of local mortality by providing a more precise and reliable “counterfactual” scenario. In Section 4.2, we make use of local mortality estimates to fully document the local and temporal evolution of the COVID-19 pandemic in Italy during the first wave.

## Literature review

In this section, we first survey the most relevant studies on excess mortality during pandemics, focusing on their methodological aspects, and then summarize the main findings of papers on mortality during the first wave of the COVID-19 pandemic in Italy.

### Excess mortality estimation: Methodological aspects

Past and recent studies have dealt with pandemics’ effects on mortality trends by estimating the excess mortality across several countries.^5^ In particular, various research analyzed the excess of deaths due to the influenza pandemic of 1918–1920, known as the Spanish flu. The majority of these studies applied intuitive approaches (see, among others, Mortara 1925; Pinnelli and Mancini 1998; Murray et al. 2006; Barro et al. 2020) or more sophisticated techniques in order to estimate excess mortality. For example, Ansart et al. (2009) used a regression model to calculate baseline and compare it with the observed mortality. The intuitive approach was also used by Luk et al. (2001) to compare the age-specific mortality during the Spanish flu with the one during other pandemics (1957 Asian Flu and 1968 Hong Kong Flu) and by Viboud et al. (2016) to analyze the pandemic of 1957-1959 in 39 countries of the world.

Within the European mortality monitoring project (EuroMOMO), a standardized approach aimed at monitoring the mortality excess due to influenza in Europe was developed.^6^ According to this approach, the mortality baseline was calculated applying a Poisson regression model, corrected for overdispersion. This approach was used by Mazick et al. (2010) that compared the all-cause deaths observed in 8 European countries during the 2009 A(H1N1) pandemic, known as the swine flu. Similar models were implemented to estimate excess mortality during the swine flu by Gran et al. (2013) for Norway and Yang et al. (2012) for Hong Kong.

Not surprisingly, as soon as official data on deaths have become available, a large number of scholars have gauged the COVID-19 impact on mortality through intuitive or more sophisticated approaches.^7^ Several scholars have developed country-level analyses that use more sophisticated approaches to estimate excess mortality due to COVID-19. For instance, Felix-Cardoso et al. (2020) used deviation from the expected value from homolog periods (DEV) and the remainder after seasonal time series decomposition (RSTS) considering total and age- and gender-specific excess mortality in five countries (England and Wales, France, Italy, Netherlands, and Portugal). Vestergaard et al. (2020), Fouillet et al. (2020), and Sinnathamby et al. (2020) employed the statistical model developed by EuroMOMO to 24 European states, to France, and England, respectively. A similar model was used by Weinberger et al. (2020) for the USA. Jiang et al. (2020) introduced a self-normalization technique to assess the trajectory of COVID-19 deaths for 30 countries. They designed a two-stage forecasting scheme to predict cumulative deaths in the USA. Li and Linton (2021) implemented a quadratic time trend model to forecast the total number of deaths (and cases) for 191 countries. Pham (2020), likewise for the US situation, dealt with the cumulative number of deaths due to the ongoing COVID-19, based on the five-parameter logistic model. Rivera et al. (2020) proposed a semiparametric method and a conventional time series method and analyzed nine US states. Vandoros (2020), for England and Wales, applied a difference-in-differences approach in order to investigate the number of deaths during the pandemic. Vieira et al. (2020) estimated the excess mortality in Portugal by comparing observed deaths with the average and respective standard deviation of the number of daily all-cause mortality in the past years to estimate mortality by age and cause. Then, they tested an ARIMA model to validate the relevant excess mortality. At a more detailed geographical level and with the use of a model that considers age group and symptom status, there is research conducted by Hauser et al. (2020) in Hubei province, China, and six regions in Europe.

Although many papers use intuitive and counterfactual approaches, only two works, to our knowledge, employ ML techniques to estimate excess mortality, though not in the context of the COVID-19 crisis. Deprez et al. (2017) investigated two classical models for estimating mortality rates in Switzerland and, by applying a regression tree boosting machine, detected different mortality models’ weaknesses. Levantesi and Pizzorusso (2019) extended the work of Deprez et al. (2017) by investigating the ability of ML to improve the accuracy of some standard stochastic mortality models, using not only decision trees but also random forest and gradient boosting.^8^

### Mortality evolution during the COVID-19 pandemic in Italy

Many studies have gauged mortality trends in Italy during the COVID-19 pandemic, applying various methodologies.

First of all, two important contributions by public institutions used the intuitive approach for estimating the impact of the COVID-19 pandemic on total resident population mortality by age and gender. The Italian National Institute of Statistics (Istat) and the National Institute of Health (ISS) have continuously monitored mortality trends in Italy, providing several reports on excess mortality.^9^ As explained above, these reports gauge the excess mortality as differences between observed mortality for all causes and the average for the same period in 2015–2019. Istat and ISS (2020a) analyzed the period January 1 to November 30, 2020, at the provincial and regional level and found around 50,000 excess deaths for the first wave of the pandemic, over 90% of which in northern Italy. Deaths were concentrated (72%) in the over 85 population. On the contrary, they found no relevant changes in the number of deaths for the period from June to September. The second institutional contribution comes from the National Institute of Social Security (INPS) (2020) that compiled a report for the first quarter of 2020, using the intuitive approach but weighting deaths for the resident population. The analysis is conducted at the provincial level with a focus on northern municipalities. The report highlighted that on April 30, the municipalities with the highest excess mortality were those located in the provinces of Bergamo, Brescia, Cremona, Lodi (Lombardy region), and Piacenza (Emilia Romagna).

Besides, many scholars deal with excess mortality at the regional level, provincial level, and municipality level using intuitive or more sophisticated approaches, above in the early phase of pandemic, when data availability was limited.

Regarding the regional level, Modi et al. (2020) use the conditional mean with a Gaussian process and synthetic control method to estimate the weekly excess of deaths for Italian regions up to April 11. They found that excess mortality is considerable in northern regions, and the cumulative deaths are higher than the deaths officially attributed to COVID-19. Investigating provincial-level trends, Ceriani and Verme (2020) compared mortality rates in 2020 with mortality rates in 2015–2019 in February to April. They found that the increase of daily mortality rates (greater for males and older) was more than 100% in mid-February in many Italian provinces (Bergamo, Cremona, Lodi, Piacenza, and Brescia), the same that experienced the highest deaths due directly to COVID-19 in the middle of March 2020. Scortichini et al. (2020) estimated the mortality excess at the provincial level with a two-stage analysis. They calculated the baseline risk by applying a quasi-Poisson regression model taking into account trends and weather conditions. Their results show an increase of deaths equal to 29.5% from the expected mortality in the period 15th of February 15th of May 2020. The majority of excess deaths (71%) were found in the northern regions (Lombardy, Emilia Romagna, and Veneto).

Given the higher incidence of COVID-19 in the north of Italy, many scholars estimated excess mortality at the municipal level, focusing on northern cities. Buonanno et al. (2020), by combining official statistics, retrospective data, and original data (i.e., obituaries and death notices), provided an estimate of excess mortality in Lombardy municipalities. They found that deaths recorded as directly due to COVID-19 constitute only half of the excess deaths verified in March. In comparison, Depalo (2021) applied partial identification to administrative data at the municipality level to estimate the number of deaths, the number of infections, and mortality rates from COVID-19 in Lombardy. He calculated that in March 2020, there were between 10,000 and 18,500 deaths, more than in the period 2015–2019.

Gibertoni et al. (2021) extended the excess mortality study on Lombardy, Emilia Romagna, and Veneto’s municipalities, differentiating for several age groups. For each subgroup, a simple linear regression on the deaths in 2015–2019 was used, and the parameters were applied to estimate the expected number of deaths in 2020. They found that the excess of deaths presents several differences among the municipalities, also within regions, and that the differences between men and women are higher in under 75.

Looking at the whole Italian territory, Del Re and Meridiani (2020) compared the excess mortality trend (calculated by subtracting a model obtained from the average deaths in 2015–2019) with the COVID-19 deaths for eight principal Italian cities considering the February to April period. They found a high excess, particularly in Brescia, and concluded that all-cause mortality excess follows the COVID-19 mortality trend. Differences between all-cause deaths and COVID-19 deaths were found in gender and age. Michelozzi et al. (2020) compared the total excess mortality across the 31 biggest Italian municipalities up to April with the deaths directly due to COVID-19. They use the intuitive approach, considering the average of the 5 previous years. They found that half of the mortality excess was due to COVID-19 deaths. Like other scholars, they observed differences between the age groups and a less relevant impact of officially COVID-19 deaths on the older people’s mortality excess. Ciminelli and Garcia-Mandicó (2020) estimated the excess mortality for 4100 northern municipalities from February 21 to March 31 by running a difference-in-differences regression model using data on mortality from 2016 as a control to mortality in 2020. They found that mortality excess was particularly high in Codogno and Alzano Lombardo (in the provinces of Lodi and Bergamo, respectively) and at the borders between Lombardy and other regions. Biggeri et al. (2020) identified spatial clusters of excess mortality for all Italian municipalities for the period January to April 2020. The mortality excess was estimated by applying a Bayesian model and considering as a baseline the average deaths 2015–2019. They found that the excess deaths are particularly diffuse in Lombardy, Piedmont, and Emilia Romagna regions. Blangiardo et al. (2020) provided a measure of the weekly excess mortality for all the Italian municipalities over the period January to April 2020 by predicting the expected mortality with a Poisson distribution and specifying a Bayesian hierarchical model on the log mortality relative risk. They found a relevant excess death in the northern regions and marked geographical differences also inside regions and between close locations.

## Data and methodology

### Data

On December 3, 2020, Istat released data on the daily number of all-cause deaths from January 1 to September 30 on all 7,904 Italian municipalities.^10^ In addition, Istat released data on the daily number of all-cause deaths for all the Italian municipalities for the years 2015–2019. We used such historical data and other variables to estimate excess death during the coronavirus outbreak in Italy. Our dependent variable is the cumulative number of deaths (per 10,000 inhabitants) calculated for three different periods: February 21 to March 31, February 21 to June 30, and February 21 to September 30.

We feed the ML models with 16 selected covariates covering aspects strongly related to deaths such as demographic, health system, economic, and contamination (air pollution) variables. This set of variables allows us to estimate the mortality trend for 2020 in the counterfactual situation, i.e., without the outbreak of coronavirus, in a more accurate way.

As COVID-19 is thought to be more lethal among men and the elderly (Dowd et al. 2020; Dudel et al. 2020), we control for the age structure, i.e., the share of men in the population, the share of those aged 65+ (overall as well as only men), and the share of those aged 80+ (overall as well as only men). Further, we control for the resident population, the overall number of deaths in the previous year, and the overall number of deaths in the period from January 1 to February 20, 2020, i.e., the 7 weeks before the coronavirus outbreak in Italy.

We also control for the number of employees as this is likely related to the heterogeneous spread of the contagion (see Ascani et al. 2020), for the share of employment in manufacturing, and for PM-10 as a measure of air quality. The latter two variables take into account that the most vulnerable people are those affected by respiratory diseases, conditions associated with high mortality in COVID-19 infection, which are more widespread in industrialized areas.^11^ For similar reasons, we also control for population density and the degree of urbanization of the municipality.

As for healthcare characteristics, we control for a dummy variable equal to 1 if there is a hospital in the municipality and another dummy variable equal to 1 if there is a hospital in at least one of the neighboring municipalities. Lastly, as the lockdown imposed after the coronavirus outbreak surely decreased the number of deaths due to road accidents, we control for the number of deaths due to road accidents in the previous year. This way, we compare municipalities with similar mortality rates due to road accidents. Table 1 reports the yearly average values of the dependent and the explanatory variables for the period 2015–2020.

**Table 1:** Descriptive statistics

	Year					
Variables	2015	2016	2017	2018	2019	2020
Number of deaths from Jan 1 to Feb 20 (per 10,000 inhabitants)	22.45	19.14	23.63	21.68	21.61	19.10
Number of deaths in the previous year (per 10,000 inhabitants)	75.65	82.97	79.29	83.37	81.02	81.48
Population	7,685.12	7,668.66	7,659.04	7,645.69	7,629.94	7,615.37
Population density (inhabitants per square kilometer)	305.23	304.72	304.49	304.11	303.69	303.45
Share of those aged 65+	23.77%	24.10%	24.42%	24.70%	25.04%	25.44%
Share of those aged 80+	7.72%	7.84%	7.97%	8.09%	8.28%	8.49%
Share of men	49.34%	49.39%	49.45%	49.53%	49.57%	49.61%
Share of men aged 65+	10.47%	10.68%	10.87%	11.05%	11.26%	11.49%
Share of men aged 80+	2.79%	2.86%	2.93%	3.00%	3.11%	3.22%
Number of employees	2,045.42	2,058.19	2,108.21	2,155.41	2,184.38	2,184.38
Share of employment in manufacturing	24.78%	24.69%	24.70%	24.35%	24.30%	24.30%
PM-10 (μg/m^3^)	28.26	28.26	25.42	26.80	24.71	24.71
Share of municipalities with a hospital	7.89%	7.81%	7.80%	7.48%	7.41%	7.41%
Share of municipalities with a hospital in at least a neighboring municipality	44.69%	44.39%	44.40%	42.68%	42.49%	42.49%
Number of deaths due to road accidents (per 10,000 inhabitants)	0.43	0.43	0.42	0.42	0.42	0.42
Number of deaths from Feb 21 to Mar 31(per 10,000 inhabitants)	15.37	14.08	14.09	14.93	14.89	21.05
Number of deaths from Feb 21 to June 30 (per 10,000 inhabitants)	45.63	43.31	44.00	44.51	45.31	55.21
Number of deaths from Feb 21 to Sept 30 (per 10,000 inhabitants)	75.34	71.55	73.40	73.34	73.77	84.58

In case of missing data for 2020, we use the 2019 value. We also control for the degree of urbanization, which is constant across years (270 municipalities are classified as large urban areas, 2,275 are classified as small urban areas, and 5,353 are classified as rural areas)

### Methodology

We use two different sets of approaches to estimate excess mortality: intuitive and ML. Estimating excess mortality requires a comparison of the observed mortality data with counterfactual mortality figures, i.e., with an estimate of the local mortality that would have been observed had the pandemic never hit Italy. This is not an easy task because, although heterogeneously, the entire country was affected by COVID-19, so that there are no “untreated” units but only “treated” ones. This implies that we cannot construct the counterfactual scenario by looking at the number of all-cause deaths in 2020 for non-affected municipalities.^12^ As a remedy for this peculiar situation, we exploit the statistical power of the time series data on all-cause deaths for the period 2015–2019 to build counterfactual mortality estimates and, in turn, derive the impact of coronavirus on local mortality.

#### The intuitive approach

The intuitive approach is straightforward: it consists of comparing the actual trend of cumulative deaths in 2020 with the trends of cumulative deaths observed in the past for the same municipality (in the previous year or for the average of previous years). In this case, we consider the average of the cumulative number of annual deaths in the period 2015–2019. This approach is easy to interpret, but it does not allow to take into account unobserved factors such as flu epidemics or climatic conditions, which can vary over time and could be highly sensitive to small changes in the input data — especially when applied at a high granularity level. The intuitive approach has recently been used in several official reports, as mentioned in Section 2.^13^

#### The ML approach

Our aim is to use ML techniques to generate a “counterfactual” scenario with predictions of what mortality figures would have been under “ordinary” conditions, i.e., if the “treatment” represented by COVID-19 would not have happened.

ML primarily deals with prediction. The recent explosion of ML in the economics literature is indeed due to the exploration of the so-called prediction policy problems (Kleinberg et al. 2015). However, recent developments have begun to exploit the predictive power of ML to also tackle causal inference research questions. In particular, Varian (2014, 2016) was the first to note that, since counterfactual building is essentially a predictive task, ML tools are, in principle, fit for the job. In a panel data setting, one could exploit the time series of pre-treatment observations to generate an artificial control group and a counterfactual trend in the no-treatment scenario. Then, by comparing the observed outcome of the treated units with their potential outcome of the counterfactual scenario, one could readily retrieve treatment effects as the difference between the two.

Following the intuition in Varian (2014, 2016), early applications have started to test the potential of this machine learning control method (MLCM) in addressing the so-called fundamental problem of causal inference, i.e., the fact that one cannot observe the potential outcome of the treated unit under the no-treatment scenario. In particular, we are aware of only seven papers employing this novel approach: Abrell et al. (2019), Benatia (2020), Benatia and de Villemeur (2019), Bijnens et al. (2019), Burlig et al. (2020), Cicala (2017), and Souza (2019). All these studies are confined to the field of energy economics and deal either with electricity markets or with the evaluation of energy efficiency policies with the exception of Bijnens et al. (2019) who look at the employment impact of the suspension of wage indexation in Belgium. Burlig et al. (2020) and Cicala (2017) have both treated and untreated units in their samples and make use of the untreated group in their counterfactual building, while Souza (2019) exploits staggered adoption to predict counterfactuals thanks to the temporal overlap of treated and not-yet-treated units. Conversely, Abrell et al. (2019), Benatia (2020), Benatia and de Villemeur (2019), and Bijnens et al. (2019) do not have an original control group but only treated units in settings with simultaneous treatment.

Importantly, Benatia (2020) is the only one to look at causal effects related to the COVID-19 crisis: it applies a neural network model to study the impact of containment measures on electricity demand. The COVID-19 pandemic is a peculiar case that lends itself well to the implementation of the ML counterfactual building method: given the worldwide spread of the contagion, it is hard to find plausible control groups as, for example, a combination of untreated units which could be used in a synthetic control approach. This makes it the ideal setting to harness the power of ML in generating an artificial counterfactual scenario.

To the best of our knowledge, this is among the first papers employing the MLCM in a setting with no control group, first proposed by Varian (2016), to evaluate the impact of a global exogenous shock affecting all the available units. Our ML predictive exercise is also closely related to the methodological strand of econometric literature, which deals with program evaluation and counterfactual building in a panel data setting (see, among others, Abadie and Cattaneo (2018) and the references therein). With respect to this literature, however, the distinctive feature of the MLCM lies in the exclusive use of pre-treatment information to generate the counterfactual scenario.

In the flourishing literature on the use of ML for policy purposes, a key concern regards two trade-offs: the one between bias and variance and the one between accuracy and interpretability. While the first is a general issue of ML techniques, the latter is distinctive of fields in which ML is used in the service of public policies that also require taking into consideration communication and accountability aspects. More complex “black box” methods tend to be both more accurate but less, if at all, interpretable. So, the choice of the appropriate technique can often fall on simpler algorithms to get more intuitive, and hence explainable, outputs at the expense of a loss in predictive accuracy. In our case, however, we are not interested in producing a transparent predictive model that clearly explains how the algorithm relates the features to the output. We just want to produce the most accurate estimates possible. This different framework is an advantage for our purpose because the trade-off between accuracy and interpretability does not impose a constraint on the selection of the techniques to employ. Thus, we opt for a mix of methods: a simpler algorithm and two black box techniques and show that, for this particular task, the simpler method performs better than the most complex ones, at least at the aggregate level.

Specifically, we adopt the following ML algorithms, LASSO, and two methods based on regression trees, random forest and stochastic gradient boosting. These algorithms are characterized by growing degrees of complexity and flexibility.^14^ LASSO is a relatively simple technique that assumes an underlying linear relationship between the outcome and the predictors. In LASSO, the model is penalized for the sum of the absolute values of the weights. The implication of this regularization is that, depending on the value of the hyperparameter λ, LASSO forces the coefficients of uncorrelated or weakly correlated predictors exactly to zero, thus performing variable selection. This makes LASSO less flexible but more interpretable than standard OLS, as it produces a sparse model in which the outcome is related only to a smaller subset of the predictors. By contrast, random forest and stochastic gradient boosting are fully non-linear methods, based on the aggregation of many decision trees.^15^ Random forest builds several different decision trees based on bootstrapped training samples and uses at each split of the trees only a random subset of the predictors as split candidates, thus decorrelating the trees from one another. The key difference with stochastic gradient boosting is that while random forest grows trees in parallel, stochastic gradient boosting grows them sequentially. Similarly to the random forest, stochastic gradient boosting is based on the aggregation and growth of many decision trees. But unlike random forest, stochastic gradient boosting does not involve bootstrap sampling, as each tree is based on the “residual” of previously grown trees, i.e., each tree is fitted on a modified version of the original dataset (Hastie et al. 2009). To make the results more comparable across the three ML methods and capture potential interactions between variables in all the selected ML models, we also include, for LASSO, all the pairwise interactions between the predictors as additional features, for a total of 256 covariates employed for this algorithm.^16^

In the ML literature, the typical routine is to randomly divide the sample in a training set, in which the model is built and tuned, and a testing set, in which its predictive power is tested through an evaluation of its out-of-sample predictive accuracy. In order to solve the other trade-off we mentioned above, the bias-variance trade-off, cross-validation on the training sample can be employed to select the best-performing values of key tuning parameters that regulate the complexity or flexibility of the algorithms and reduce the risk of overfitting.

Bearing the above in mind, for each of the three different periods (February 21 to March 31, February 21 to June 30, and February 21 to September 30), we implement the following process: (i) we split the 2015–2018 pooled dataset into a training sample, made up of 80% of the municipalities, and a testing sample, which consists of the remaining 20%; (ii) we train and tune all the algorithms on the training sample, on which we perform a tenfold cross-validation to select the best-performing tuning hyperparameters of each algorithm;^17^ (iii) we test how well the algorithms perform in predicting observed mortality on unseen data, i.e., on the 2015–2018 testing sample; (iv) we test model performance on the entire 2019 sample and show that algorithm performance is stable over time and that all the ML methods perform, on average, better than the commonly adopted intuitive method; (v) we repeat this routine on the pooled 2015–2019 data, to train the models on as much data as possible so as to maximize the accuracy gains; (vi) we use the models built on the 2015–2019 dataset to predict, for the 2020 sample, estimates of local mortality in a “no-COVID” scenario; and (vii) we derive municipality-level excess deaths for all the municipalities by subtracting the ML counterfactual estimates from the observed 2020 mortality data released by Istat. Municipality-level excess death estimates are relevant, especially because they can be considered as one of the best possible proxies to gauge the local magnitude of the COVID-19 impact. The underlying assumptions of our methodology are exactly the same of those underlying the intuitive method, namely, that (i) we are in a relatively static environment, at least on average (Burlig et al. 2020), i.e., mortality trends are generally not very volatile over time, with the exception of extraordinary events such as a war or a pandemic, which seems reasonable in our context; (ii) the difference between the observed and counterfactual mortality outcomes is due to the total impact of the exogenous “shock” represented by the COVID-19 pandemic.^18^ Below we will provide descriptive and factual evidence to demonstrate the credibility of both assumptions.

Although our dataset is a panel of Italian municipalities, in our benchmark models, we consider the data as pooled, following Andini et al. (2018). In fact, we treat each municipality–year pair as if it was a single observation. We do not deem this as a problematic aspect as we focus on a relatively short time period, whereas such a choice could entail issues if some of the variables we use (either the outcome or the predictors) were to exhibit drastic changes over time within the same municipality, and this is unlikely to happen in a short time span. In any case, in the results section, we also report the predictive performance of an ML model, which explicitly incorporates the longitudinal component of the sample via the inclusion of municipality and year dummies and show that there are no predictive gains with respect to our benchmark estimates. The descriptive statistics reported in Table 1 show that the year-by-year variation in mortality data and key predictors is rather low, reinforcing the validity of assumption (i) in our context. Finally, an in-time placebo test performed on the year 2019, discussed in Section 4.2, will serve to underpin the plausibility of both assumptions.

Importantly, we apply our random splitting on *municipalities*, not on *municipality–year* pairs. Going for the latter would make the same municipality appear in the training set in 1 year (e.g., 2016) but in the testing set in another year (say, 2018). To the extent that the predictors do not change or vary slowly over time, this would produce downward-biased estimates of the mean squared error (MSE), because the testing data would not truly be “unseen” data, but data very similar to their counterparts for the corresponding municipality in the years that appear in the training sample. By splitting on municipalities, instead, we are sure that the training and testing samples do not share any municipality in common, and the same municipality can only appear either in the training or in the testing set for the entire time span.

Finally, for the sake of comparison of ML performance with that of a simpler, linear, and widely adopted method, we also report the 2019 predictive performance of a standard OLS model run on the full 2015–2018 sample.

## Estimates

### Predictive power of all methods used

We begin the empirical analysis by examining the forecasting performance of all methods used. Given the presence of approaches different from ML, we have opted for evaluating the performance of the various approaches in the estimation of the number of deaths per 10,000 inhabitants in an “ordinary” year. We select 2019 as the “ordinary” year and use data from 2015 to 2018 to test the predictive power of all methods. For forecast evaluation, we employ the mean squared error (MSE), i.e., the mean of the squared value of the prediction errors, as the main model selection approach. However, as MSE is very sensitive to outliers, we also report the mean absolute error (MAE), i.e., the mean of the absolute value of the errors. These two measures are our metrics to conduct a comparative analysis of predictive performances. Table 2 reports the results for all the sets of methods at the dates of March 31, June 30, and September 30.

**Table 2:** A comparison of predictive accuracy across the different methods

Method	MSE, March 31, 2019	MAE, March 31, 2019	MSE, June 30, 2019	MAE, June 30, 2019	MSE, September 30, 2019	MAE, September 30, 2019
Panel A: performance on all municipalities						
Intuitive (historical average)	216.59	8.70	665.63	15.75	1,082.81	20.32
Intuitive (past year)	351.95	10.37	1,104.24	19.46	1,679.86	24.60
OLS	179.51	8.30	558.50	14.98	932.00	19.77
LASSO	**178.03**	8.09	**550.33**	14.56	**900.81**	18.86
Random forest	179.97	8.16	557.23	14.63	914.23	18.94
Boosting	179.01	8.20	555.13	14.82	902.96	19.15
Panel B: performance by population size						
	< 2,000 inhabitants (3,457 municipalities)					
Intuitive (historical average)	450.36	1,4.31	1,379.64	26.06	2,240.62	33.70
Intuitive (past year)	733.24	16.78	2,309.04	32.26	3,488.76	40.64
OLS	370.56	13.48	1,140.86	24.15	1,877.86	31.15
LASSO	**369.81**	13.33	**1,134.83**	23.93	1,846.62	30.68
Random forest	374.36	13.52	1,152.28	24.20	1,882.25	31.09
Boosting	370.09	13.34	1,138.49	24.08	**1,839.38**	30.79
	Between 2,000 and 5,000 inhabitants (2,030 municipalities)					
Intuitive (historical average)	56.91	5.89	180.05	10.41	297.08	13.35
Intuitive (past year)	91.13	7.38	275.80	12.92	446.85	16.43
OLS	48.42	5.57	159.15	9.90	285.05	13.41
LASSO	**46.25**	5.34	**150.80**	9.53	257.80	12.61
Random forest	46.36	5.35	150.95	9.57	**254.32**	12.49
Boosting	47.27	5.42	155.45	9.75	264.38	12.80
	Between 5,000 and 50,000 inhabitants (2,265 municipalities)					
Intuitive (historical average)	16.69	3.12	53.39	5.66	88.81	7.27
Intuitive (past year)	26.16	3.84	78.15	6.85	130.74	8.78
OLS	16.66	3.25	61.89	6.24	124.30	8.98
LASSO	14.73	2.99	50.86	5.50	89.91	7.37
Random forest	**14.47**	2.96	**48.52**	5.35	**86.08**	7.20
Boosting	16.70	3.25	57.50	5.95	101.84	7.99
	⩾ 50,000 inhabitants (146 municipalities)					
Intuitive (historical average)	2.56	1.30	9.09	2.44	**13.69**	2.93
Intuitive (past year)	4.14	1.59	14.17	2.96	24.87	3.85
OLS	4.85	1.78	25.81	4.08	61.42	6.31
LASSO	2.96	1.35	14.16	2.97	26.43	4.00
Random forest	**2.27**	1.20	**8.62**	2.31	16.33	3.12
Boosting	4.58	1.73	19.48	3.50	37.20	4.79

Best-performing method in terms of MSE in bold

The crucial insight from Panel A of Table 2 is that all ML techniques and OLS always perform better than the intuitive approach. On average, MSE is reduced by up to 17.8% (February 21 to March 31), 17.4% (February 21 to June 30), 16.8% (February 21 to September 30), compared to using the mainstream approach adopted by several Italian and international institutions. This is the key result of the paper. The pattern is qualitatively consistent when considering MAE. A closer look at individual performances reveals that LASSO is the best-performing ML algorithm, both in terms of MSE and MAE. Random forest and boosting fare worse, but not by much, and still lead to a substantial improvement in precision over the intuitive approaches. When considering MSE, LASSO is followed by boosting, but when looking at MAE, random forest outperforms boosting. These rankings remain consistent for all three periods under scrutiny.^19^ While the superior performance of a linear method like LASSO is somewhat unusual compared to other works in the ML literature, it can be explained by the nature of the predictive problem and the limited number of observations and predictors. In such circumstances, it can be expected that simpler algorithms can match the performance of more complex methods (de Blasio et al. 2020).^20^ Indeed, this is why the OLS performance is comparable to that of the ML routines. Note that, in a few instances, OLS is even more accurate than some of the ML algorithms; however, it is never the best-performing technique, and there is always an ML technique that is superior. Yet, it fares always better than the intuitive approaches, so one may be willing to trade-off a lower MSE/MAE for a more straightforward methodology that does not require the complexity of ML techniques.

Panel B of Table 2 reports the performance of all estimators by population size. First and foremost, the magnitude of the prediction error is inversely proportional to the municipality size. While such sharp inter-class heterogeneity may seem striking, this is actually not surprising when taking into account that the dependent variable is defined as the number of deaths per 10,000 inhabitants and that the variability of growth rates of any variable in small municipalities is substantially higher. Second, concerning model performance, there is also some heterogeneity depending on population size. For example, LASSO and OLS tend to perform worse for large municipalities. The intuitive method, instead, works fine for big cities, where it becomes competitive with ML performance but is imprecise and too coarse for small- and medium-sized municipalities. This confirms our research hypothesis that this method, being based on unconditional averaging, can be very sensitive to outliers and small changes in the input data, features that matter most when looking at smaller municipalities than larger ones, where mortality figures tend to be much more stable. Indeed, municipalities above the 50,000 population cutoff, above which the intuitive method becomes competitive with ML, account for only 1.85% (146/7,904) of the total number of Italian municipalities. For more than 98% of our sample, all the ML methods perform far and above the official approach. As in Panel A of Table 2, OLS is never the most accurate technique. Besides, it tends to become less and less competitive as municipality size gets bigger and as the time span increases, with an especially pronounced drop in accuracy for municipalities above 50,000 inhabitants. This is a serious limitation of the OLS algorithm with respect to the ML algorithms, which prove instead to be comparatively more stable across all population cutoffs and time periods under scrutiny.

In the following section, we show the excess death mortality estimates for 2020 as computed by the best-performing algorithm for each of the four population cutoffs (see Panel B of Table 2).

### Deriving excess death figures during the first wave of the COVID-19 pandemic

**Fig. 1:**
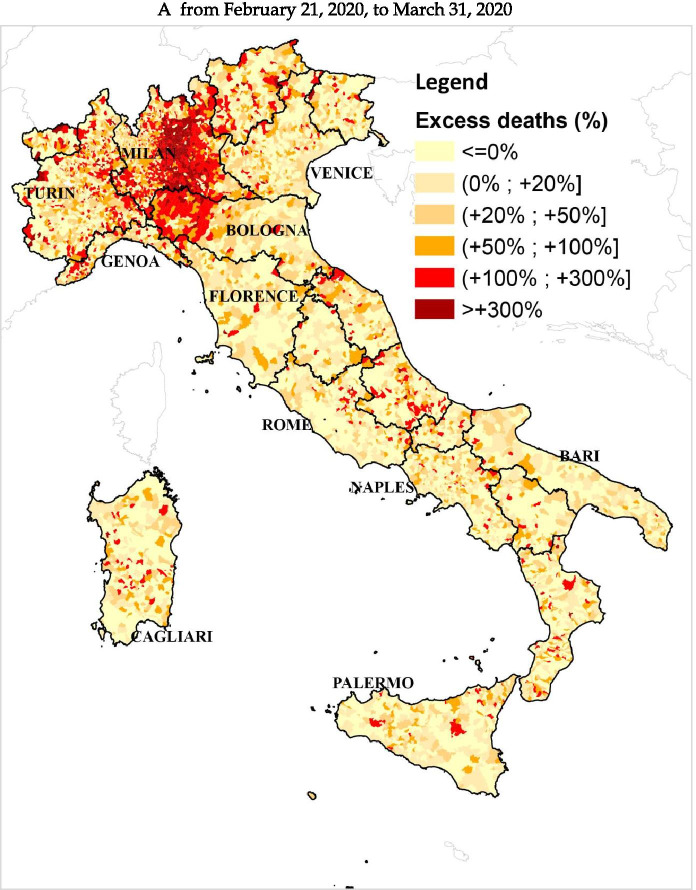

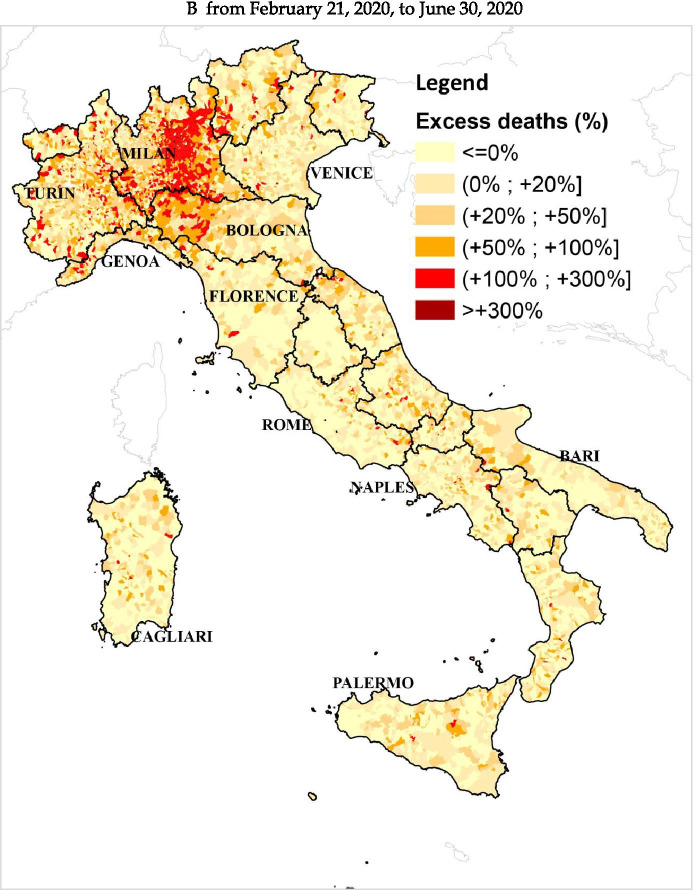

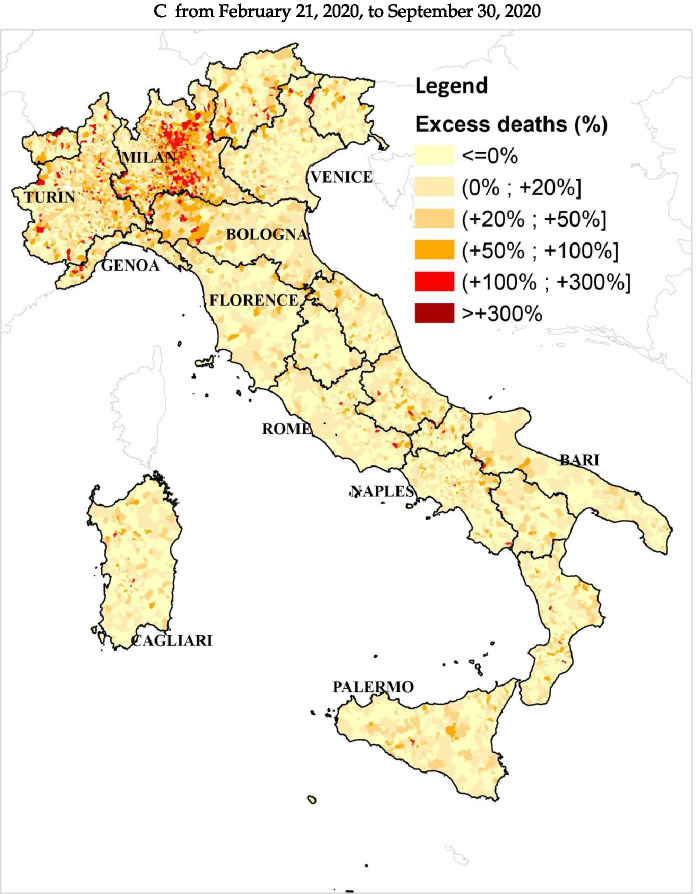
Percentage of municipal excess deaths detected from February 21, 2020, to September 30, 2020, with respect to the counterfactual scenario estimated via ML techniques. **A** From February 21, 2020, to March 31, 2020 (note: excess mortality estimates for the north of Italy, 23,603; official number of COVID-19 deaths, 11,011. Gap between these estimates on March 31, 12,592). **B** From February 21, 2020, to June 30, 2020 (note: excess mortality estimates for the north of Italy, 40,001; official number of COVID-19 deaths, 29,752. Gap between these estimates on June 30, 10,249). **C** From February 21, 2020, to September 30, 2020 (note: excess mortality estimates for the north of Italy, 39,362; official number of COVID-19 deaths, 30,580. Gap between these estimates on September 30, 8,782)

**Fig. 2:**
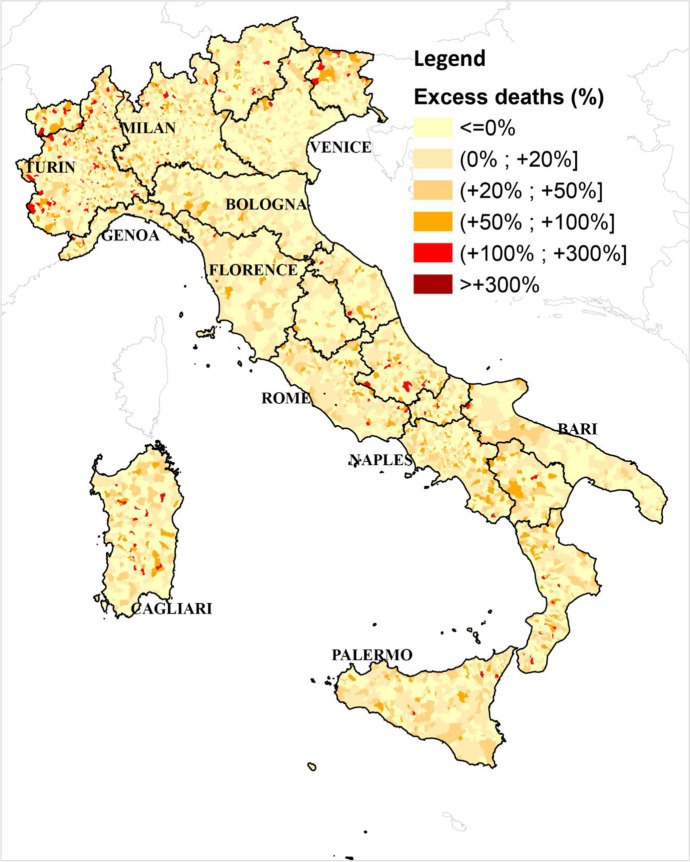
Percentage of municipal excess deaths detected from February 21, 2019, to June 30, 2019, with respect to predicted deaths estimated via ML techniques. Note: Excess mortality estimates (measurement error) at the country level: 1,365

The excess mortality estimates from all-cause deaths are not uniform throughout Italy, as can be seen in Fig. 1. Significant differences emerge across and within geographical areas. The excess mortality estimates obtained via ML techniques for the period from February 21 to September 30 are particularly high in various northern municipalities. In particular, at the end of March (Fig. 1A), 393 municipalities, mostly concentrated in Lombardy, record an estimated excess of deaths over 300%. Further, 1,251 municipalities (28.57% of northern municipalities) record a percentage of excess mortality higher than 100%. They are mostly located in the Lombardy region and the western part of the Emilia Romagna region. Many of these territories are located in the provinces that record the highest number of infections up to that date, namely, the provinces of Milan, Bergamo, Brescia, Cremona, and Lodi (Lombardy), and the provinces of Piacenza, Parma, and Reggio Emilia (Emilia Romagna). This is in line with the results of Ceriani and Verme (2020), Modi et al. (2020), and Biggeri et al. (2020), which analyzed the period February to April. Several municipalities in the provinces of Cuneo, Alessandria (Piedmont), and Imperia (Liguria) constituted clusters with excess mortality higher than 100%, and similar clusters were in the provinces of Trento (Trentino-Alto Adige) and Pesaro-Urbino (Marche), consistently with the findings of Blangiardo et al. (2020). On June 30 (Fig. 1B), only very few municipalities in Bergamo and Brescia provinces recorded an estimated excess higher than 300%. Lombardy region continued to record the highest number of municipalities with an estimated excess of deaths over 100%, while in the provinces of Piacenza, Parma, and Reggio Emilia (Emilia Romagna), the majority of municipalities recorded an excess between 50 and 100% compared to the counterfactual scenario. A decrease in excess of deaths below 100% occurs for several municipalities in Cuneo and Imperia provinces. On September 30 (Fig. 1C), the number of municipalities with an excess higher than 100% in the Lombardy region is sensibly lower (212), but many municipalities (574) still record an excess of deaths above 50%. Clusters of municipalities with estimated excess mortality above 100% were observed over time in other northern regions affected by the spread of the virus, such as Piedmont and Liguria. The decrease observed in the previous period in Cuneo and Imperia provinces continues in the subsequent period, and on September 30, the municipalities with a mortality excess between 50% and 100% are considerably less in comparison to the end of June. In the other geographical areas, the estimated mortality excess was much lower for the whole period. From February 21 to March 31 (Fig. 1A), the majority of municipalities recorded estimated excess mortality under 50%, with very few municipalities with excess mortality over 100%. On June 30 (Fig. 1B), the number of municipalities with an excess of deaths between 20% and 100% was much lower than the previous snapshot, and in the central and southern Italy, municipalities with higher excess mortality deaths seemed to be less widespread compared to the snapshots on March 31. The substantially lower excess death figures in central and southern areas are also confirmed by the results of Blangiardo et al. (2020) and Scortichini et al. (2020) who focus on similar time spans. By September 30 (Fig. 1C), there are no visible excess mortality clusters in these areas. Fig. 2, Table 3, and Table 4 in the Appendix clearly show that the excess death estimates in the center-south of Italy can be considered in line with what one would expect to happen in an “ordinary” year (2019 in our case), while the observed trend in the north of Italy is extraordinarily abnormal.^21^ The consistency of the estimates with the geographical evolution of COVID-19 cases confirms that the estimated mortality excess from all-cause deaths is connected, both directly and indirectly, to the COVID-19 pandemic. A note of caution pertains to small municipalities where even sporadic deaths could determine large variations in percentage terms, especially when considering a short time interval such as February 21 to March 31.^22^

Our study allows drawing preliminary conclusions about the effects of the pandemic on the spatio-temporal evolution of the local excess deaths during the first wave. The estimated excess mortality for some municipalities between February 21 and June 30 is lower than the values estimated for the period February 21 to March 31. As pointed out by Istat and ISS (2020b), the worst-affected provinces in the first phase of the crisis start to experience a decrease of deaths in the second half of April. This decrease may be connected with demographic changes, behavioral factors, pandemic control measures, and also with the improved diagnostic capacity and a minor pressure on the national health system (Istat and ISS, 2020a). However, by June 30, the estimated excess mortality remains high in many municipalities in northern Italy and abnormal clusters of excess mortality persist in well-defined areas. The decrease in the estimates of excess mortality emerges even more when looking at the whole period from February 21 to September 30. Although small clusters of municipalities with high excess deaths persist, above all in the Lombardy region, the local mortality excess is lower and substantially less widespread. The period June to September is considered a transition period in the spread of the COVID-19 infection, and according to the Istat and ISS (2020a), during the summer months, the mortality trend was in line with the average observed in the previous years. Therefore, the cumulative mortality excess observed in our map at the end of September in several northern municipalities is entirely due to the excess of the all-cause deaths during the first phase of the pandemic.

By aggregating the municipality excess mortality estimates in the north of Italy, we find an increase of 23,603 deaths compared to the counterfactual scenario for the period from February 21 to March 31, 2020. This estimate is in line with the figures obtained by INPS, Istat, and ISS (2020a) by employing the intuitive approach and suggests that the “official” number of deaths directly due to COVID-19 (11,011) might be severely underestimated. However, the gap between our excess mortality estimates (39,362) and the “official” number of deaths directly due to COVID-19 in Northern Italy (30,580) becomes slightly smaller by the end of September. This finding suggests that, despite delays in reporting the attribution of deaths to COVID-19 and irrespective of potential harvesting effects, the tracking capacity of Italian authorities improved over the course of the pandemic.

## Conclusions

In this work, we propose a more sophisticated approach to produce local estimates of excess mortality during the first wave (February to September 2020) of the COVID-19 pandemic in Italy. Specifically, as COVID-19 is a major global exogenous “shock” affecting all municipalities, we adopt a novel MLCM to build a more plausible counterfactual scenario of mortality figures for 2020 in the absence of the pandemic. We show that the ability of ML methods to build the artificial control group in a “no-COVID-19” scenario outperforms the mainstream intuitive approach adopted by Italian institutions, with particularly sizable predictive gains at the more granular level of small- and medium-sized municipalities. Importantly, our methodological improvements do not depend on any additional identifying condition, i.e., our assumptions are exactly the same as those underpinning the intuitive method.

After showing that these methodologies improve performance by up to 17.8%, we build a municipality-level dataset of the first-wave excess death mortality figures by comparing counterfactual and observed mortality data in each municipality. This dataset, which is shared jointly with the paper, is intended to be available to the general public and to researchers interested in investigating local determinants and territorial factors that may have contributed to the rapid and heterogeneous spread of the first wave of the pandemic across Italy, as well as for evaluating policy responses at a local level and compare spatio-temporal differences with the evolution of the ongoing second wave. We hope our methodological contribution will lead to further refinements of the current approaches targeted at estimating mortality during the current pandemic and, in turn, to a broader understanding of the spread of the virus in Italy and the efficacy of the policies adopted to contain its impacts.

Our methodological framework could be extended to other countries and, possibly, to the entire European Union, to study the temporal and spatial evolution of the first as well as the ongoing second wave. Besides, we emphasize that there is room for more research as, in principle, the inclusion of a richer set of covariates, a comprehensive integration of spatial autocorrelation patterns (such as the inclusion of additional features capturing economic links across municipalities), or the adoption of other ML methods, may further increase the statistical accuracy of our approach. Nonetheless, our main conclusion remains that the intuitive approach can lead to imprecise excess mortality estimates, especially when applied at a high spatial granularity, and should be possibly replaced by more advanced data-driven techniques.

### Supplementary Information

ESM 1 (XLSX 1165 kb)

## Data Availability

Resource site (in Italian), https://www.stimecomunalicovid19.com; dataset, available for download in the “Dataset” window of the resource site.

## Appendix

**Table 3:** Share of excess deaths “observed” in 2019 and in 2020 by population size

	Share of municipalities with excess deaths above 50%		Share of municipalities with excess deaths above 100%		Share of municipalities with excess deaths above 300%	
	2019	2020	2019	2020	2019	2020
Overall	8.81%	21.64%	2.32%	9.50%	0.05%	0.37%
Less than 2,000 inhabitants	17.01%	26.57%	5.12%	11.86%	0.12%	0.66%
Inhabitants ⩾ 2,000 and < 5,000	4.53%	20.98%	0.30%	8.71%	0.00%	0.15%
Inhabitants ⩾ 5,000 and < 50,000	0.71%	15.69%	0.00%	7.05%	0.00%	0.13%
More than 50,000 inhabitants	0.00%	5.44%	0.00%	2.04%	0.00%	0.00%

**Table 4:** Share of excess deaths “observed” in 2019 and in 2020 by geographic and population size

	Share of municipalities with excess deaths above 50%		Share of municipalities with excess deaths above 100%		Share of municipalities with excess deaths above 300%	
	2019	2020	2019	2020	2019	2020
North						
Overall	9.21%	32.48%	2.83%	16.12%	0.09%	0.66%
Less than 2,000 inhabitants	17.38%	34.75%	5.94%	18.61%	0.20%	1.14%
Inhabitants ⩾ 2,000 and < 5,000	3.98%	33.22%	0.35%	14.97%	0.00%	0.27%
Inhabitants ⩾ 5,000 and < 50,000	0.59%	28.64%	0.00%	13.39%	0.00%	0.25%
More than 50,000 inhabitants	0.00%	14.29%	0.00%	6.12%	0.00%	0.00%
Center-south						
Overall	8.32%	8.15%	1.68%	1.25%	0.00%	0.00%
Less than 2,000 inhabitants	16.48%	15.20%	3.96%	2.48%	0.00%	0.00%
Inhabitants ⩾ 2,000 and < 5,000	5.22%	5.49%	0.22%	0.78%	0.00%	0.00%
Inhabitants ⩾ 5,000 and < 50,000	0.83%	1.49%	0.00%	0.09%	0.00%	0.00%
More than 50,000 inhabitants	0.00%	1.02%	0.00%	0.00%	0.00%	0.00%

**Table 5:** Variable importance ranking of the random forest algorithm (2015–2018 training sample for the period February 21 to September 30)

Variable	Increase in node purity
Share of those aged 80+	5,633,789
Share of those aged 65+	4,576,871
Share of men aged 80+	3,579,953
Share of men aged 65+	3,367,116
Population density (inhabitants per square kilometer)	2,521,136
Population	2,508,496
Number of employees	2,385,304
Number of deaths from Jan 1 to Feb 20 (per 10,000 inhabitants)	1,887,305
Share of men	1,853,599
PM-10 (μg/m^3^)	1,601,275
Share of employment in manufacturing	1,463,918
Number of deaths in the previous year (per 10,000 inhabitants)	1,449,038
Degree of urbanization	384,249.6
Share of municipalities with a hospital in at least a neighboring municipality	220,769.3
Number of deaths due to road accidents (per 10,000 inhabitants)	140,360.1
Share of municipalities with a hospital	23,915.52

## Footnotes

1 Official data on coronavirus reported cases are released only at the provincial level (the number of infected people) or at the regional level (the number of coronavirus deaths).

2 On December 3, 2020, the Italian National Institute of Statistics (Istat) released the number of daily certified deaths for the period January 1 to September 3, 2020, for all 7,904 Italian municipalities.

3 Excess mortality is the number of deaths from all causes during a crisis above and beyond what we would have expected to see under “normal” conditions (see Roser et al. 2020).

4 However, there is some empirical evidence that suggests that COVID-19 was already present in northern Italy at the end of January (see Cerqua and Di Stefano 2021).

5 The following influenza pandemics occurred in the twentieth and twenty-first centuries: the Spanish flu in 1918–1920, the Asian flu in 1957–1958, the Hong Kong flu in 1968, and the Swine flu in 2009 (World Health Organization).

6 For further information: https://www.euromomo.eu/.

7 This literature review provides a comprehensive but not exhaustive review of the studies on the COVID-19 impact on mortality. Country-level analysis with intuitive methodologies is also conducted by Modig et al. (2021) for Sweden, Nogueira et al. (2020) for Portugal**,** and Stang et al. (2020) for Germany.

8 ML algorithms applied to research questions related to COVID-19 are used by Bonacini et al. (2021), Dandekar and Barbastathis (2020), and Magri and Doan (2020).

9 ISS is the main institution for research, control, and technical scientific advice on public health in Italy.

10 Due to the creation of Mappano as a new administrative unit in 2017 and to the lack of mortality data for all years, we cannot analyze 6 municipalities: Borgaro Torinese, Caselle Torinese, Leini, Mappano, and Settimo Torinese. Besides, as 2020 is a leap year, we decided to ignore the deaths that occurred on February 29 for reasons of comparability with data from previous years.

11 Employment data come from the Statistical Register of Active Enterprises (ASIA) archive, which covers the universe of firms and employees of industry and services. PM-10 data are taken from the European Environment Agency: http://aidef.apps.eea.europa.eu/?source=%7B%22query%22%3A%7B%22match_all%22%3A%7B%7D%7D%2C%22display_type%22%3A%22tabular%22%7D. PM-10 data is from 573 monitoring stations distributed across the Italian territory. We employ the kriging spatial interpolation to impute the PM-10 average yearly value for each municipality.

12 In principle, one could make use of municipalities of southern regions or the islands that were less affected by the spread of the contagion. But, apart from the fact that, in the absence of reliable and complete data on the spread of the contagion, it would be difficult to set an objective threshold for belonging to this control group, these municipalities would be so different under almost every aspect from the more severely hit northern municipalities to make any attempt of counterfactual building using these controls futile.

13 In Italy, it was adopted by: - Istat and ISS report at the Italian regional and provincial level https://www.istat.it/it/files//2020/12/Rapp_Istat_Iss.pdf); - INPS report at the Italian provincial level with a focus on northern Italian municipalities (https://www.inps.it/docallegatiNP/Mig/Dati_analisi_bilanci/Nota_CGSA_mortal_Covid19_def.pdf) - SISMG to monitor the situation of daily deaths for the elderly population (over 65 years) for Italian cities with more than 250,000 inhabitants (https://repo.epiprev.it/index.php/download/andamento-della-mortalita-giornaliera-sismg-nelle-citta-italiane-in-relazione-allepidemia-di-covid-19-report-1-febbraio-2-maggio-2020-settimo-rapporto/?wpdmdl=1626&refresh=5ee8e7ed5223a1592322029). - European Statistical Office (Eurostat) provided estimates of the excess mortality for the European Union and EFTA countries comparing all-cause deaths with deaths between January 2016 to October 2019 https://ec.europa.eu/eurostat/statistics-explained/index.php?title=Excess_mortality_-_statistics

14 Here, we only provide an overview of the main differences between the three methodologies. For an exhaustive description and more technical details on each of these methods, please refer to Hastie et al. (2009).

15 Although boosting can be applied to other methods than the decision tree, the one based on decision trees is by far one of the most popular versions.

16 Unlike LASSO, random forest and boosting by default take into account all the possible non-linearities and interactions between the features.

17 The hyperparameters we select via tenfold cross-validation are the following: for LASSO, the parameter *λ* which controls the shrinkage penalty; for the random forest, the parameter *m*, i.e., the number of features randomly sampled as candidates at each split (for the number of trees to grow, instead, we use the default value of 1,000); and for boosting, the shrinkage parameter representing the learning rate, the number of trees to fit and the minimum number of observations in the terminal nodes of the tree. Cross-validation is used by running different models with several candidate values (or combinations of values, in the case of boosting) for all these parameters.

18 Concerns about the plausibility of the stable unit treatment value assumption (SUTVA) for the potential outcomes under the COVID-19 treatment (but not for potential outcomes under the *no-treatment* scenario, as we generate the no-COVID scenario using only pre-COVID data) are legitimate in our setting (see Rubin 1980). Indeed, under the COVID-19 treatment, the potential outcomes of a given municipality are a function of the COVID-19 spread on other municipalities. Yet, the spatial spread of the contagion among municipalities does not threaten the reliability of our estimates. In fact, our goal is to estimate the *gross* effect of COVID-19 on each municipality, i.e., the total number of deaths directly or indirectly due to the pandemic (compared to a no-pandemic scenario), irrespective of how the number of infections and, thus, deaths, is affected by spillovers from nearby municipalities, which are unavoidably part of the very definition of a pandemic. Finally, note that these SUTVA-related issues equally apply to the intuitive approach.

19 Furthermore, for interested readers, Table 5 in the Appendix presents the absolute variable importance ranking generated by the random forest algorithm for 2015–2018 training sample of the February 21 to September 30 period.

20 For the sake of comparison with a longitudinal model, the predictive performance of a random forest model enriched with a longitudinal component, applied on the 2019 dataset for the February 21 to September 30 period, was the following: 923.73 (MSE) and 19.12 (MAE). Both performance metrics are inferior to the corresponding ones of the baseline pooled version of random forest model for the same period (see Panel A of Table 2). We also added spatial lags of all control variables (computed as the average value of all border municipalities) with the idea that information about neighboring municipalities should decrease the estimation error. However, the addition of spatially lagged covariates reduced the MSE only by 0.8%. In light of this very small increase, we opted for using the more parsimonious models reported in the main text.

21 In the Appendix (Figure 2), we report a map of a placebo analysis conducted on the year 2019, where we have used data from 2015 to 2018 to estimate excess mortality figures during an “ordinary” year. Finding large excess mortality clusters in 2019, before COVID-19 hit Italy, would cast doubts on the plausibility of our approach. As the reader can see, this in-time falsification test shows no excess mortality clusters whatsoever in 2019, as one would expect, since there was no pandemic. Besides, we find that there is no spatial autocorrelation in the estimation error of this 2019 test (Moran’s I index of 0.015), whereas we find a strong spatial correlation for the excess mortality estimates from February 21, 2020, to June 30, 2020 (Moran’s I index of 0.362). This again suggests that the spatial clustering is not due to specific features of our estimation approach but indeed to the arrival of the pandemic. Such evidence lends credibility to both assumptions underpinning our approach. Lastly, Tables 3 and 4 in the Appendix compare the excess deaths “observed” in 2019 and in 2020 by population size and by geographical area.

22 Some small municipalities in the center-south of Italy exhibit excess mortality higher than 100% by the end of March. However, such large effects, which are mostly due to random variation amplified by their low population figures, are short-lived as they are averaged away when considering longer time periods (see Fig. 1B and C).

## References

[CR1] Abadie A, Cattaneo MD (2018). Econometric methods for program evaluation. Ann Rev Econ.

[CR2] Abrell J, Kosch M, Rausch S (2019) How effective was the UK carbon tax? A machine learning approach to policy evaluation. A Machine Learning Approach to Policy Evaluation (April 15, 2019)*.* CER-ETH–Center of Economic Research at ETH Zurich Working Paper, 19, 317

[CR3] Andini M, Ciani E, de Blasio G, D'Ignazio A, Salvestrini V (2018). Targeting with machine learning: an application to a tax rebate program in Italy. J Econ Behav Organ.

[CR4] Ansart S, Pelat C, Boelle PY, Carrat F, Flahault A, Valleron AJ (2009). Mortality burden of the 1918–1919 influenza pandemic in Europe. Influenza Other Respir Viruses.

[CR5] Ascani A, Faggian A, Montresor S (2020) The geography of COVID-19 and the structure of local economies: the case of Italy. J Reg Sci, first published online: 20 November 2020 10.1111/jors.12510 PMC7753650 33362296

[CR6] Barro RJ, Ursua JF, Weng J (2020) The coronavirus and the great influenza epidemic. Lessons from the “Spanish flu” for the coronavirus’s potential effects on mortality and economic activity. NBER Working Paper Series, 26866

[CR7] Benatia D (2020) Reaching new lows? The pandemic’s consequences for electricity markets. USAEE Working Paper no. 20-454

[CR8] Benatia D, de Villemeur EB (2019) Strategic reneging in sequential imperfect markets. CREST Working Papers 2019-19

[CR9] Biggeri A, Lagazio C, Catelan D, Barbone F, Braga M (2020). A municipality-level analysis of excess mortality in Italy in the period January-April 2020. Epidemiol Prev.

[CR10] Bijnens G, Karimov S, Konings J (2019) Wage indexation and jobs. A machine learning approach. VIVES Discussion Paper no. 82 10.1007/s10645-023-09418-y PMC9932419 36820314

[CR11] Blangiardo M, Cameletti M, Pirani M, Corsetti G, Battaglini M, Baio G (2020). Estimating weekly excess mortality at sub-national level in Italy during the COVID-19 pandemic. PLoS One.

[CR12] Bonacini L, Gallo G, Patriarca F (2021). Identifying policy challenges of COVID-19 in hardly reliable data and judging the success of lockdown measures. J Popul Econ.

[CR13] Buonanno P, Galletta S, Puca M (2020). Estimating the severity of COVID-19: evidence from the Italian epicenter. PLoS One.

[CR14] Burlig F, Knittel CR, Rapson D, Reguant M, Wolfram C (2020). Machine learning from schools about energy efficiency. J Assoc Environ Resour Econ.

[CR15] Ceriani L, Verme P (2020) Excess mortality as a predictor of mortality crises: the case of COVID-19 in Italy. GLO Discussion Paper Series 618

[CR16] Cerqua A, Di Stefano R (2021) When did coronavirus arrive in Europe? Statistical Methods and Applications, first published online: 20 May 2021 10.1007/s10260-021-00568-4 PMC8136371 34035795

[CR17] Ceylan Z (2020). Estimation of COVID-19 prevalence in Italy, Spain, and France. Sci Total Environ.

[CR18] Cicala, S. (2017). Imperfect markets versus imperfect regulation in US electricity generation. NBER Working Paper no. 23053

[CR19] Ciminelli G, Garcia-Mandicó S (2020). COVID-19 in Italy: an analysis of death registry data. J Public Health.

[CR20] Dandekar R, Barbastathis G (2020) Quantifying the effect of quarantine control in COVID-19 infectious spread using machine learning. medRxiv. 10.1101/2020.04.03.20052084

[CR21] de Blasio G, D’Ignazio A, Letta M (2020) Predicting corruption crimes with machine learning. A study for the Italian municipalities. DiSSE Sapienza Working Paper Series, no. 16/2020

[CR22] Del Re D, Meridiani P (2020) Monitoring the COVID-19 epidemics in Italy from mortality data. medRxiv. 10.1101/2020.05.07.20092775

[CR23] Depalo D (2021). True Covid-19 mortality rates from administrative data. J Popul Econ.

[CR24] Deprez P, Shevchenko PV, Wüthrich MV (2017). Machine learning techniques for mortality modeling. Eur Actuar J.

[CR25] Dowd JB, Andriano L, Brazel DM, Rotondi V, Block P, Ding X, … Mills MC (2020) Demographic science aids in understanding the spread and fatality rates of COVID-19. Proc Natl Acad Sci U S A, 117(18), 9696-9698 10.1073/pnas.2004911117 PMC7211934 32300018

[CR26] Dudel C, Riffe T, Acosta E, van Raalte A, Strozza C, Myrskylä M (2020). Monitoring trends and differences in COVID-19 case-fatality rates using decomposition methods: contributions of age structure and age-specific fatality. PLoS One.

[CR27] Felix-Cardoso J, Vasconcelos H, Rodrigues P, Cruz-Correia R (2020) Excess mortality during COVID-19 in five European countries and a critique of mortality analysis data. medRxiv. 10.1101/2020.04.28.20083147

[CR28] Fouillet A, Pontais I, Caserio-Schönemann C (2020). Excess all-cause mortality during the first wave of the COVID-19 epidemic in France, March to May 2020. Euro Surveillance.

[CR29] Ghislandi, S., Muttarak, R., Sauerberg, M., Scotti, B. (2020). News from the front: estimation of excess mortality and life expectancy in the major epicenters of the COVID-19 pandemic in Italy. medRxiv. 10.1101/2020.04.29.20084335

[CR30] Gibertoni D, Adja KYC, Golinelli D, Reno C, Regazzi L, Lenzi J, ..., Fantini MP (2021) Patterns of COVID-19 related excess mortality in the municipalities of Northern Italy during the first wave of the pandemic. Health & Place 67:102508 10.1016/j.healthplace.2021.102508 PMC7834600 33476843

[CR31] Gran JM, Kacelnik O, Grjibovski AM, Aavitsland P, Iversen BG (2013). Counting pandemic deaths: comparing reported numbers of deaths from influenza A (H 1 N 1) pdm09 with estimated excess mortality. Influenza Other Respir Viruses.

[CR32] Hastie T, Tibshirani R, Friedman J (2009). The elements of statistical learning: data mining, inference, and prediction.

[CR33] Hauser A, Counotte MJ, Margossian CC, Konstantinoudis G, Low N, Althaus CL, Riou J (2020). Estimation of SARS-CoV-2 mortality during the early stages of an epidemic: a modelling study in Hubei, China, and six regions in Europe. PLoS Med.

[CR34] Inps (2020) Analisi della mortalità nel periodo di epidemia da Covid-19. 20 May 2020, Rome. Available at https://www.inps.it/docallegatiNP/Mig/Dati_analisi_bilanci/Nota_CGSA_mortal_Covid19_def.pdf

[CR35] Istat, ISS (2020a). Decessi per il complesso delle cause. Periodo gennaio-novembre 2020. 30 December 2020, Rome. Available at https://www.istat.it/it/files//2020/12/Rapp_Istat_Iss.pdf

[CR36] Istat, ISS (2020b) Impatto dell’epidemia COVID-19 sulla mortalità totale della popolazione residente periodo gennaio-maggio 2020. 9 July 2020, Rome. Available at www.istat.it/it/files//2020/07/Rapp_Istat_Iss_9luglio.pdf

[CR37] Jiang F, Zhao Z, Shao X (2020) Time series analysis of COVID-19 infection curve: a change-point perspective. Journal of Econometrics, first published online 30 July 2020 10.1016/j.jeconom.2020.07.039 PMC7392157 32836681

[CR38] Kleinberg J, Ludwig J, Mullainathan S, Obermeyer Z (2015). Prediction policy problems. Am Econ Rev.

[CR39] Leon DA, Shkolnikov VM, Smeeth L, Magnus P, Pechholdová M, Jarvis CI (2020). COVID-19: a need for real-time monitoring of weekly excess deaths. Lancet.

[CR40] Levantesi S, Pizzorusso V (2019). Application of machine learning to mortality modeling and forecasting. Risks.

[CR41] Li S, Linton O (2021). When will the Covid-19 pandemic peak?. J Econ.

[CR42] Luk J, Gross P, Thompson WW (2001). Observations on mortality during the 1918 influenza pandemic. Clin Infect Dis.

[CR43] Magri L, Doan NAK (2020) First-principles machine learning modelling of COVID-19. arXiv preprint arXiv: 2004.09478

[CR44] Mazick A, Gergonne B, Wuillaume F, Danis K, Vantarakis A, Uphoff H (2010). Higher all-cause mortality in children during autumn 2009 compared with the three previous years: pooled results from eight European countries. Eurosurveillance.

[CR45] Michelozzi P, de’Donato F, Scortichini M, Pezzotti P, Stafoggia M, De Sario M, ... Davoli M (2020) Temporal dynamics in total excess mortality and COVID-19 deaths in Italian cities. BMC Public Health, 20(1): 1-8 10.1186/s12889-020-09335-8 PMC7426899 32795276

[CR46] Modi C, Boehm V, Ferraro S, Stein G, Seljak U (2020) How deadly is COVID-19? A rigorous analysis of excess mortality and age-dependent fatality rates in Italy. medRxiv. 10.1101/2020.04.15.20067074

[CR47] Modig, K., Ahlbom, A., Ebeling, M. (2021). Excess mortality from COVID-19. weekly excess death rates by age and sex for Sweden and its most affected region. European Journal of Public Health 31(1):17–22 10.1093/eurpub/ckaa218 PMC7717265 33169145

[CR48] Mortara G (1925). La salute pubblica in Italia durante e dopo la guerra.

[CR49] Murray CJ, Lopez AD, Chin B, Feehan D, Hill KH (2006). Estimation of potential global pandemic influenza mortality on the basis of vital registry data from the 1918–20 pandemic: a quantitative analysis. Lancet.

[CR50] Nogueira PJ, de Araújo Nobre M, Nicola PJ, Furtado C, Carneiro AV (2020). Excess mortality estimation during the COVID-19 pandemic: preliminary data from Portugal. Acta Medica Port.

[CR51] Pham H (2020). On estimating the number of deaths related to COVID-19. Mathematics.

[CR52] Pinnelli A, Mancini P (1998). Mortality peaks in Italy in the late 19^th^ and early 20^th^ centuries: trends by age and sex. Eur J Popul.

[CR53] Rivera R, Rosenbaum JE, Quispe W (2020). Excess mortality in the United States during the first three months of the COVID-19 pandemic. Epidemiol Infect.

[CR54] Roser M, Ritchie H, Ortiz-Ospina E, Hasell J (2020) Coronavirus pandemic (COVID-19)*.* Our world in data. Available at: https://ourworldindata.org/coronavirus. Accessed 24 Jan 2021

[CR55] Rubin DB (1980). Comment on “randomization analysis of experimental data: the fisher randomization test” by D. Basu. J Am Stat Assoc.

[CR56] Scortichini M, Dos Santos RS, De’Donato F, De Sario M, Michelozzi P, Davoli M, ... Gasparrini A (2020) Excess mortality during the COVID-19 outbreak in Italy: a two-stage interrupted time-series analysis. Int J Epidemiol, 49(6), 1909–1917 10.1093/ije/dyaa169 PMC7665549 33053172

[CR57] Sinnathamby MA, Whitaker H, Coughlan L, Lopez Bernal J, Ramsay M, Andrews N (2020) All-cause excess mortality observed by age group and regions in the first wave of the COVID-19 pandemic in England. Euro Surveillance 25(28) 10.2807/1560-7917.ES.2020.25.28.2001239 PMC7376843 32700669

[CR58] Souza M (2019) Predictive counterfactuals for treatment effect heterogeneity in event studies with staggered adoption. SSRN*.*10.2139/ssrn.3484635

[CR59] Stang A, Standl F, Kowall B, Brune B, Böttcher J, Brinkmann M, ... Jöckel KH (2020) Excess mortality due to COVID-19 in Germany. J Inf Secur, 81(5), 797-801 10.1016/j.jinf.2020.09.012 PMC7501062 32956730

[CR60] Vandoros S (2020) Excess mortality during the COVID-19 pandemic: early evidence from England and Wales. Soc Sci Med, 258 10.1016/j.socscimed.2020.113101 PMC7836531 32521411

[CR61] Varian HR (2014). Big data: new tricks for econometrics. J Econ Perspect.

[CR62] Varian HR (2016). Causal inference in economics and marketing. Proc Natl Acad Sci.

[CR63] Vestergaard LS, Nielsen J, Richter L, Schmid D, Bustos N, Braeye T, ... Mølbak K (2020) Excess all-cause mortality during the COVID-19 pandemic in Europe–preliminary pooled estimates from the EuroMOMO network, March to April 2020. Eurosurveillance, 25(26), 2001214 10.2807/1560-7917.ES.2020.25.26.2001214 PMC7346364 32643601

[CR64] Viboud C, Simonsen L, Fuentes R, Flores J, Miller MA, Chowell G (2016). Global mortality impact of the 1957–1959 influenza pandemic. J Infect Dis.

[CR65] Vieira A, Peixoto VR, Aguiar P, Abrantes A (2020). Rapid estimation of excess mortality during the COVID-19 pandemic in Portugal-beyond reported deaths. J Epidemiol Glob Health.

[CR66] Weinberger DM, Chen J, Cohen T, Crawford FW, Mostashari F, Olson D, Viboud C (2020). Estimation of excess deaths associated with the COVID-19 pandemic in the United States, March to May 2020. JAMA Internal Medicine.

[CR67] Yang L, Chan KP, Cowling BJ, Chiu SS, Chan KH, Peiris JSM, Wong CM (2012). Excess mortality associated with the 2009 pandemic of influenza A (H1N1) in Hong Kong. Epidemiol Infect.

